# Liver progenitor cell-driven liver regeneration

**DOI:** 10.1038/s12276-020-0483-0

**Published:** 2020-08-14

**Authors:** Juhoon So, Angie Kim, Seung-Hoon Lee, Donghun Shin

**Affiliations:** Department of Developmental Biology, McGowan Institute for Regenerative Medicine, Pittsburgh Liver Research Center, University of Pittsburgh, Pittsburgh, PA 15260 USA

**Keywords:** Regeneration, Reprogramming

## Abstract

The liver is a highly regenerative organ, but its regenerative capacity is compromised in severe liver diseases. Hepatocyte-driven liver regeneration that involves the proliferation of preexisting hepatocytes is a primary regeneration mode. On the other hand, liver progenitor cell (LPC)-driven liver regeneration that involves dedifferentiation of biliary epithelial cells or hepatocytes into LPCs, LPC proliferation, and subsequent differentiation of LPCs into hepatocytes is a secondary mode. This secondary mode plays a significant role in liver regeneration when the primary mode does not effectively work, as observed in severe liver injury settings. Thus, promoting LPC-driven liver regeneration may be clinically beneficial to patients with severe liver diseases. In this review, we describe the current understanding of LPC-driven liver regeneration by exploring current knowledge on the activation, origin, and roles of LPCs during regeneration. We also describe animal models used to study LPC-driven liver regeneration, given their potential to further deepen our understanding of the regeneration process. This understanding will eventually contribute to developing strategies to promote LPC-driven liver regeneration in patients with severe liver diseases.

## Regenerative medicine: Understanding regeneration in liver disease

A deeper understanding of the biology of liver progenitor cells (LPCs) could give clinicians more effective tools for reversing the effects of liver disease. LPCs are precursors of the liver cells known as hepatocytes, and naturally appear when the tissue is injured or diseased. Juhoon So, Donghun Shin and colleagues at the University of Pittsburgh, USA, have reviewed the current state of knowledge of LPC biology. They explore the mechanisms by which LPCs are generated and activated in response to trauma. These molecular pathways could offer useful drug targets.The authors also point out that excessive LPC activity can promote liver damage and tumorigenesis. As a consequence, any regenerative medicine approach based on LPCs must be coupled with effective strategies for promoting the efficient maturation of these cells into healthy hepatocytes.

## Introduction

The liver is an essential and multifunctional organ in vertebrates. It consists of hepatocytes and biliary epithelial cells (BECs) that are differentiated from common progenitor cells called hepatoblasts during development. Hepatocytes, a major cell type in the liver, detoxify various metabolites, regulate glucose and lipid metabolism, synthesize serum proteins, and secrete bile. BECs form the biliary network that transports bile from hepatocytes to the gallbladder^1^. Upon food ingestion, bile is released from the gallbladder into the duodenum and helps absorb fats in the gut.

The liver is also a highly regenerative organ. It is able to restore its mass and function after injury. Depending on the source of regenerating hepatocytes, there are two modes of liver regeneration: hepatocyte- and LPC-driven liver regeneration^2,3^. For instance, upon two-third partial hepatectomy, the remaining hepatocytes proliferate to restore the resected liver mass^4^ (hepatocyte-driven regeneration). On the other hand, when hepatocyte proliferation is compromised, BECs are able to dedifferentiate into liver progenitor cells (LPCs), also known as oval cells. Then, these LPCs later differentiate into hepatocytes^2,3^ (LPC-driven regeneration). In addition to this BEC-to-LPC dedifferentiation, hepatocytes can dedifferentiate into LPCs and later differentiate back into hepatocytes^5^.

Given that liver diseases have been a major health concern due to their high prevalence and poor long-term clinical outcome, the regenerative potential of the liver is especially important to note. Globally, approximately two million deaths per year are caused by severe liver diseases, including viral hepatitis, liver cirrhosis, and liver cancer^6^. Currently, liver transplantation is the only curative option for these life-threatening diseases; however, the shortage of donor livers limits this option. Thus, patients with severe liver diseases often die while waiting for a donor liver^6,7^. Given that LPC-driven liver regeneration is prevalent in severe liver injury settings, augmenting this regeneration mode should be clinically beneficial to patients with severe liver diseases.

In this review, we focus mainly on LPC-driven liver regeneration by examining recent findings and exploring current knowledge about the activation, origin, and role of LPCs during liver regeneration. We also provide information on the clinical significance and therapeutic potential of LPCs.

## Features of LPCs

Hepatocyte-driven liver regeneration is achieved by the proliferation of preexisting hepatocytes^4^, whereas LPC-driven liver regeneration is achieved by the proliferation and subsequent differentiation of LPCs^2,3^. Thus, LPC-driven liver regeneration is important in severe liver injury settings accompanied by impaired hepatocyte proliferation. In LPC-driven liver regeneration, hepatocytes or BECs first dedifferentiate into LPCs following LPC proliferation, and the LPCs then differentiate into hepatocytes^1,8^.

**Fig. 1:**
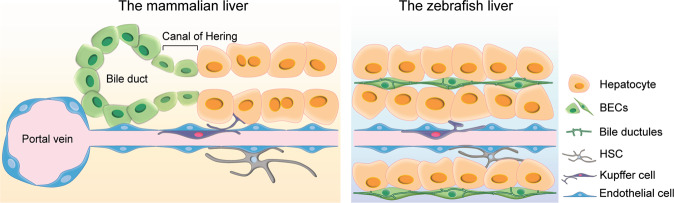
The structure of the liver in mammals and zebrafish. The liver consists of hepatocytes, BECs, Kupffer cells, HSCs, and endothelial cells. LPCs are thought to arise near bile ductules, also known as the canals of Hering, which are positioned between the bile duct and hepatocytes. In the zebrafish liver, most BECs make up bile ductules.

Over time, LPCs have been variously named in rodent and human studies as oval cells, hepatic progenitor cells, liver stem cells, ductular reactions, or atypical ductular cells^9^. They were first described as oval cells in rats due to their large nuclear-to-cytoplasm ratio and oval-shaped nuclei^10^. Anatomically, LPCs have been suggested to reside within the canals of Hering, also known as intrahepatic bile ductules, which are positioned between the bile duct and hepatocytes^1^ (Fig. 1). Although LPCs are not observed in the normal adult liver, they appear and expand in response to severe or chronic liver injury^2,3^. LPCs express both hepatocyte (KRT8, KRT18, and albumin)^11–13^ and BEC (KRT7, KRT19, EpCAM, and SOX9) markers^11,14–16^. Depending on injury settings, LPCs also express the hepatoblast marker α-fetoprotein (AFP)^17^, hematopoietic markers, such as CD34, CD90, CD133, c-Kit, CXCR4, and Sca1 (refs. ^12,18–21^), or the neuronal marker NCAM^22^. The various expression of these markers in LPCs suggests their progenitor features and heterogeneous nature. Recently, single-cell RNA sequencing analyses of EpCAM^+^ hepatic cells isolated from mice fed a 3,5-diethoxycarbonyl-1,4-dihydrocollidine (DDC) diet further revealed the heterogeneity of LPCs^23,24^.

## The molecular mechanisms of LPC activation

Extensive LPC activation and expansion are detected in patients with various liver diseases, including alcoholic or nonalcoholic liver disease^25^, chronic viral hepatitis^26,27^, and cholestatic hepatitis^28^, all of which cause a massive loss of hepatocytes in the liver. LPC activation, also referred to as ductular reactions, involves the proliferation and expansion of LPCs in the periportal regions in the liver, thereby increasing the number of bile ductules. Additionally, LPC activation involves macrophage infiltration, extracellular matrix remodeling, and myofibroblast activation^29,30^.

The mechanisms of LPC activation have been extensively investigated using animal models of chronic liver injury. Hepatocyte-specific deletion of the inhibitor kappa B kinase with Alb-Cre mice inhibited hepatocyte proliferation and induced apoptosis^31^. Dying hepatocytes in these mice produced hedgehog ligands that promote the outgrowth of LPCs and myofibroblasts^31^. Hepatocyte-specific deletion of survivin with Alb-Cre mice also induced hepatocyte apoptosis and inflammation, eliciting LPC activation^32^. These findings support that the extensive loss of hepatocytes and the impairment of hepatocyte proliferation induce LPC activation. Furthermore, it was recently reported that YAP and mTORC1 signaling are important for LPC activation. YAP and mTORC1 signaling positively regulate the growth of BEC-derived organoids in vitro and the proliferation of BECs and LPCs in mice^24^. TET1-mediated epigenetic remodeling through YAP signaling was also recently reported to positively control LPC activation^33^.

Additionally, macrophages are known to associate with LPC activation during regeneration. Dying hepatocytes and their debris are engulfed and removed by Kupffer cells, which are the resident macrophages in the liver. These macrophages secrete tumor necrosis factor (TNF)-like weak inducer of apoptosis (TWEAK), a member of the proinflammatory TNF family, and TWEAK binds to its receptor FGF-inducible 14 (Fn14) expressed on LPCs^30^. Fn14 was increasingly expressed on LPCs in chronic hepatitis C and other human liver diseases^30^. Moreover, overexpression of TWEAK in hepatocytes stimulated LPC proliferation through the nuclear factor-κB (NF-κB) signaling pathway^30,34^.

TNFα^35^, interferon-γ (IFNγ)^36^, and interleukin-6 (IL-6) (refs. ^35,37^) signaling are also known to control LPC activation. Moreover, other inflammatory-related proteins, including cyclooxygenase-2 (ref. ^38^), lymphotoxin beta^39,40^, and galectin-3 (ref. ^41^), are reported to regulate LPC activation. Growth factor signaling pathways, such as HGF/c-Met^42,43^, TGF-β^42,44,45^, FGF7 (ref. ^46^), and VEGF^47^ signaling, are also involved in LPC activation and expansion (Table 1).

**Table 1:** List of molecules involved in LPC activation and their expressing cell types.

Molecular function or signaling pathway	Molecule	Expressing cell type	References
Cytokine	IFNγ	LPC	^36,40^
	IL-6	Inflammatory cell	^35,37,40^
DNA demethylation	TET1	LPC	^33^
FGF signaling	FGF7	Thy1^+^ mesenchymal cell	^46^
	FGFR2	LPC	^46^
	FGFBP1	LPC	^46^
HGF/c-Met signaling	c-Met	LPC	^43^
	HGF	HSC	^42^
IL-6/STAT3 signaling	SOCS3	LPC, inflammatory cell, hepatocyte	^37^
Integrin signaling	Galectin-3	LPC, hepatocyte, macrophage	^41^
mTOR signaling	Ribosomal S6	LPC	^116^
NF-kB signaling	NF-kB	LPC, hepatocyte	^31^
TGF signaling	GDF11	HSC	^45^
	TGF-β	HSC	^42^
	β2-spectrin	LPC	^44^
	TGF-β type II receptor	LPC	^44^
Shh signaling	Gli2	LPC, hepatocyte	^31^
	Ihh	Hepatocyte	^31^
TNF signaling	Cox2	LPC, Kupffer cell, endothelial cell	^38^
	LTβ	LPC, inflammatory cell	^26,40^
	FN14	LPC	^34^
	TWEAK	Macrophage	^30^
	TNFα	LPC, inflammatory cell	^35,40^
VEGF signaling	VEGFA	Hepatocyte	^47^
	VEGFC	Hepatocyte	^47^
	VEGFR1	LPC	^47^
	VEGFR3	LPC	^47^

## Origins of LPCs

**Fig. 2:**
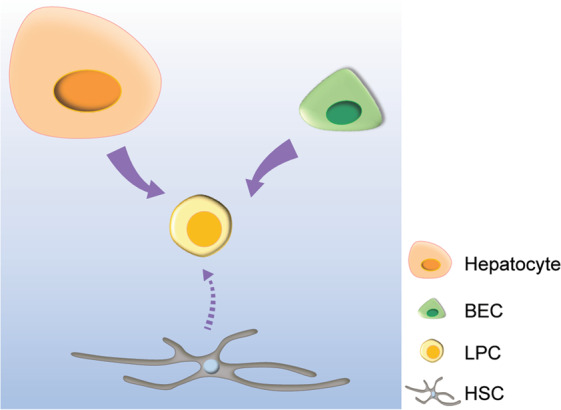
Origins of LPCs. BECs and hepatocytes can give rise to LPCs in various liver injury settings. HSCs might be an additional source of LPCs in certain liver injury settings.

Despite the absence of LPC-specific markers that are expressed in LPCs but not in BECs, BECs were hypothesized to be the origin of LPCs due to their phenotypical similarity and locational contiguity^48^ (Fig. 2). This was later validated by lineage tracing. By genetically labeling nearly all hepatocytes in mice, the Grompe group showed that BECs in mice fed a DDC diet for 2 weeks contributed to LPCs^5^. The same group also showed, by tracing the lineage of *Sox9*^+^ BECs with Sox9-CreERT2 mice, that BECs gave rise to LPCs in DDC diet, choline-deficient, ethionine-supplemented diet (CDE), and CCl_4_ injury models^49^. By tracing the lineage of BECs with Krt19-CreERT mice, it was also shown that BECs contribute to LPCs in the DDC and CDE models^50,51^. Although these lineage-tracing studies have validated BECs as the origin of LPCs, the activated LPCs in the studies did not differentiate into hepatocytes. Later, the fact that LPCs originate from BECs was also confirmed in liver injury models in which LPCs significantly contribute to hepatocytes^52^. In mice with β1-integrin knocked down in all hepatocytes, thus blocking hepatocyte proliferation, BEC lineage tracing revealed the significant contribution of BECs to hepatocytes in several liver injury models, including DDC^52^. Hepatocyte-specific overexpression of p21 combined with the liver injury models also exhibited a significant contribution of BECs to hepatocytes^52^. BEC-to-LPC dedifferentiation was also observed in zebrafish^53–55^. Upon the severe loss of hepatocytes in zebrafish larvae by pharmacogenetic means, BECs dedifferentiated into LPCs and subsequently differentiated into hepatocytes. In this zebrafish model, nearly all hepatocytes in the recovered larvae originated from BECs^53^.

In addition to BECs, lineage-tracing studies revealed hepatocytes as an additional source of LPCs^5,51,56^ (Fig. 2). By labeling nearly all hepatocytes in mice, the Grompe group showed that hepatocytes in mice fed a DDC diet for 6, but not 2, weeks contributed to LPCs^5^. Once the liver injury was gone, these hepatocyte-derived LPCs reverted to hepatocytes^5^. These data suggest that hepatocytes can contribute to the LPC population by undergoing metaplasia in chronic liver injury settings. Supporting this hepatocyte origin, overexpression of constitutively active YAP1 (ref. ^57^) or Notch^56^ in mature hepatocytes converted the hepatocytes to LPCs. Moreover, inhibiting YAP^23^ or Notch^56^ signaling in hepatocytes suppressed their conversion to LPCs in the DDC model. Hepatocyte-to-LPC dedifferentiation was also observed in zebrafish^58^. In *Tg(fabp10a:pt-β-catenin)* zebrafish larvae, which overexpress a stable form of β-catenin in hepatocytes, a subset of hepatocytes dedifferentiated into LPCs and later differentiated into hepatocytes^58^, as observed in the mice fed a DDC diet^5^. Although some lineage-tracing studies suggested hepatic stellate cells (HSCs) as an origin of LPCs and regenerated hepatocytes^59–62^, other lineage-tracing studies showed no conversion of HSCs to LPCs or hepatocytes in multiple liver injury settings^50,63^, raising uncertainty regarding HSCs as an origin of LPCs (Fig. 2).

## The beneficial role of LPCs: their differentiation into hepatocytes

**Fig. 3:**
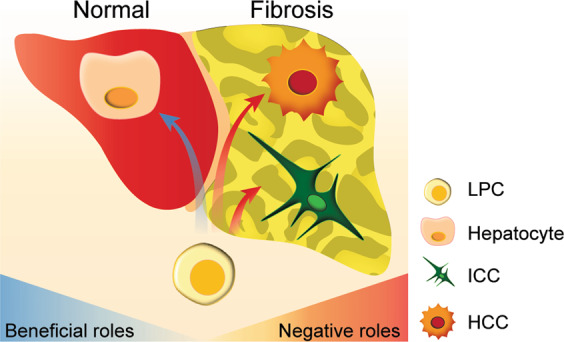
The beneficial and negative roles of LPCs. LPCs play a beneficial role in liver regeneration/recovery by differentiating themselves into hepatocytes, whereas they play negative roles by contributing to liver fibrosis and tumor formation, such as HCC and ICC.

Studies with diseased human livers have suggested that BECs dedifferentiate into LPCs and that LPCs can differentiate into hepatocytes^11,64–67^ (Fig. 3). Supporting these human studies, lineage-tracing studies in mice^52,68–71^ and zebrafish^53–55^ have demonstrated that in severe liver injury settings, BECs first dedifferentiate into LPCs and subsequently differentiate into hepatocytes. For the initial mouse lineage-tracing studies, several BEC-specific, inducible Cre lines, such as Opn-CreERT2, Krt19-CreERT, Hnf1b-CreERT2, and Sox9-CreERT2, were used to trace the fate of BECs in DDC- and CDE-mediated liver injury models. While no contribution of BECs to hepatocytes was observed in the DDC model, a few hepatocytes (<2.5%) originated from BECs in the CDE model^5,50,72–74^. This low percentage of contribution of BECs to hepatocytes raised a question about the significance of LPC-driven regeneration in liver regeneration. However, several groups recently showed a significant contribution of BECs to hepatocytes in severe liver injury settings in which hepatocyte proliferation is greatly compromised. To block hepatocyte proliferation, *Mdm2* (ref. ^70^), β1-integrin^52^, or β-catenin^68^ was deleted specifically in hepatocytes, or p21 was overexpressed in hepatocytes^52^. In addition to these genetic blocks of hepatocyte proliferation, long-term chronic liver injury elicited the natural impairment of hepatocyte proliferation, thereby inducing the differentiation of BEC-derived LPCs into hepatocytes^69,71^. As previously mentioned in the section on LPC origins, hepatocytes as well as BECs can give rise to LPCs, and hepatocyte-derived LPCs appear to revert to hepatocytes during recovery^5^. For the zebrafish lineage-tracing studies, the BEC-specific, inducible Cre line, *Tg(Tp1:CreERT2)*, was used in the complete hepatocyte-ablation model. Upon severe hepatocyte loss, BECs dedifferentiate into LPCs, and subsequently, LPCs differentiate into either hepatocytes or BECs^53–55^. Intriguingly, suppressing LPC differentiation in the zebrafish model impaired liver regeneration and recovery^75,76^, suggesting the beneficial effect of LPC differentiation on liver recovery.

Given the beneficial effect of promoting LPC differentiation, elucidation of the molecular mechanisms of LPC differentiation has been attempted. Liver-specific *c-Met* knockout mice fed a DDC diet exhibited a significant reduction in the number of A6^+^/EpCAM^−^ hepatocyte-like cells compared to *c-Met* wild-type mice, suggesting that HGF/c-Met signaling may regulate the differentiation of LPCs into hepatocytes during regeneration^43^. In addition, treatment with iloprost, a laminin signaling inhibitor, increased a LPC-derived hepatocyte population in the CDE model, suggesting that LPC microenvironment may control its differentiation^72^. Notch and Wnt/β-catenin signaling are also involved in LPC differentiation^29^. Wnt3a secreted from macrophages induces the expression of Numb, which inhibits Notch signaling, in LPCs, thereby promoting the differentiation of LPCs into hepatocytes. Jag1 expressed in myofibroblasts increases Notch signaling in LPCs, thereby promoting the differentiation of LPCs into BECs^29^. In addition to these mouse studies, zebrafish studies revealed additional players that control LPC differentiation. Suppressing BMP signaling inhibited the differentiation of LPCs into hepatocytes, and this LPC differentiation defect was also observed in *smad5* and *tbx2b* zebrafish mutants^75,76^. Suppressing Hdac1 activity derepressed *sox9b* expression, thereby inhibiting the differentiation of LPCs into hepatocytes^75,76^.

## The negative roles of LPCs: fibrosis and liver cancer

Hepatic fibrosis, the formation of an abnormally large amount of scar tissue in the liver, occurs in most chronic liver diseases. As a response to liver injury, quiescent HSCs are activated to become profibrogenic myofibroblasts^77^. This HSC activation is associated with LPC activation^78^. A correlation between the number of LPCs and the severity of fibrosis in chronic liver diseases^25,27,78–80^ suggests that LPCs may promote hepatic fibrosis (Fig. 3). T helper type 1 (Th1) cells produce IFNγ, which regulates LPC proliferation^81^. BALB/c mice deficient in Th1 signaling fed a CDE diet exhibited reduced LPC proliferation and fibrosis compared to C57Bl/6 mice, which have normal Th1 signaling. Supplementation of IFNγ increased both LPC number and fibrosis in the CDE model^36^. In addition, TWEAK, which induces LPC activation, was associated with liver fibrosis^82^. Administration of TWEAK induced both LPC activation and collagen expression^83^, whereas *Fn14* knockout mice fed a CDE diet exhibited reduced LPC proliferation and collagen expression^34^. These results together suggest that LPCs could drive liver fibrosis (Fig. 3).

Liver cancer is the sixth most commonly diagnosed cancer and the fourth leading cause of cancer death worldwide in 2018, accounting for approximately 841,000 new cases and 782,000 deaths annually^6^. Of primary liver cancer cases, 75–85% are hepatocellular carcinoma (HCC), and 10–15% are intrahepatic cholangiocarcinoma (ICC). LPC activation is known to be associated with pathological scarring processes and appears to contribute to liver tumor formation^84^. LPC markers, such as KRT7, KRT19, OV6, and EpCAM, are detected in HCCs^85,86^. More importantly, inhibition of LPC proliferation in chronically injured mouse livers reduced tumor development^38,87,88^. Additionally, functional genomics analysis of human HCCs revealed a Notch-associated signature in one-third of human HCCs^89^. Persistent Notch signaling activation in mouse livers induced features of human hepatocarcinogenesis, including dysplasia and HCC^89^. Given that Notch signaling regulates cancer stem cells during hepatocarcinogenesis^90^, these results together suggest a role for LPCs in HCC formation (Fig. 3).

Given its biliary features, ICC was thought to originate from BECs. Indeed, lineage-tracing studies confirmed the BEC origin^91^. However, ectopic and persistent activation of Notch and AKT signaling in hepatocytes generated ICC, revealing hepatocytes as an additional origin of ICC^92,93^. Moreover, neomorphic mutations of IDH1 or IDH2 that acquire abnormal activity to convert α-ketoglutarate to 2-hydroxyglutarate lead to ICC formation by inhibiting the differentiation of LPCs into hepatocytes^94^. An additional type of primary liver carcinoma that is of interest is combined or mixed hepatocellular cholangiocarcinoma (cHCC-CCA). It has been considered that cHCC-CCA originates from LPCs, which can differentiate into both hepatocytes and BECs^95^. cHCC-CCA also exhibits stem or progenitor features with downregulation of the hepatocyte differentiation program and a commitment to the biliary lineage^96^. These findings together suggest that LPCs contribute to ICC formation regardless of their origin.

## Animal models for LPC research

Given the high cost and ethical issues of human studies, animal models have been used to study human liver diseases. Among them, rodents are widely used because of their remarkable genetic similarity to humans^97^. LPCs were first identified in rats^98^, and their activation was primarily investigated in rat models of chronic liver injury in which hepatocyte proliferation was compromised^99^. In this model, hepatocyte necrosis was induced by injecting d-galactosamine, and AFP^+^ LPCs were observed in the periportal area^99^. LPC activation and proliferation were also observed in the Solt–Farber liver injury model in which 2-acetylaminofluorene was administered to rats followed by two-third partial hepatectomy^100,101^.

Given the advantage of genetic manipulation in mice, mouse models have been widely used for LPC research^97^. Mice fed a DDC diet are used to study metabolic liver diseases, sclerosing cholangitis, and biliary fibrosis^102^. Mice fed a CDE diet exhibit steatosis, inflammation, LPC activation and expansion, portal fibrosis, and HCC^103^. CCl_4_ is also widely used as a hepatic toxin that induces chronic liver injury. Repeated injection of CCl_4_ causes centrilobular necrosis followed by a wound-healing process^97^. It also induces LPC activation and expansion, fibrosis, and cirrhosis^69,97,104^. Additionally, administration of thioacetamide induces chronic inflammation, LPC activation and expansion, fibrosis, cirrhosis, and liver cancer^71,105,106^. Since all these liver toxins induce LPC activation and expansion, these mouse models have been used to investigate the mechanisms of LPC activation and expansion.

As previously mentioned in the section on the beneficial role of LPCs, additional mouse models for BEC/LPC-driven liver regeneration in which BECs significantly contribute to regenerating hepatocytes have recently been established. In these mouse models, liver toxins, such as CDE and DDC, were used to induce liver injury, and hepatocyte proliferation was additionally suppressed^52,68^. Using the hepatocyte-specific p21-overexpressing model, the positive role of TET1 in LPC-driven liver regeneration was recently reported^33^. These mouse models will allow one to better understand the molecular mechanisms of LPC-driven liver regeneration, particularly LPC differentiation.

In addition to rodent models, zebrafish have relatively recently been used for LPC research due to their small size, which allows for simple chemical treatment. Various zebrafish liver injury models, including one-third partial hepatectomy^107,108^, ethanol treatment^55,109^, oncogene-induced liver cancers^110–112^, and hepatocyte ablation^53–55^, have been established. In particular, the hepatocyte-ablation model has been used to study LPC-driven liver regeneration. Our group developed the *Tg(fabp10a:CFP-NTR)* zebrafish line that expresses nitroreductase (NTR) under the hepatocyte-specific *fabp10a* promoter. Since NTR converts the nontoxic prodrug metronidazole (Mtz) into a cytotoxic drug, Mtz treatment specifically ablates hepatocytes in *Tg(fabp10a:CFP-NTR)* fish. Upon extensive hepatocyte loss, BECs dedifferentiate into LPCs, and LPCs subsequently differentiate into hepatocytes, thereby leading to full liver recovery^53^. Using this model, we recently reported that BMP signaling regulates LPC-driven liver regeneration through Tbx2a and Id2a^75^ and that bromodomain and extraterminal proteins (BET), Hdac1, Kdm1a, Sox9b, and Notch3 regulate LPC-driven liver regeneration^76,113,114^. Using the same zebrafish model, two other groups also reported that mTORC1 signaling regulates LPC-driven liver regeneration^115,116^.

In the *Tg(fabp10a:CFP-NTR)* model, LPC-driven liver regeneration occurs robustly and rapidly. Although this rapid regeneration has allowed us to identify small molecules that impair liver regeneration^53^, it is not suitable for identifying compounds that promote LPC-driven liver regeneration. Our group has recently established a new zebrafish liver injury model for LPC-driven liver regeneration in which LPCs slowly differentiate into hepatocytes^58^. In this model, *Tg(fabp10a:pt-β-catenin)* zebrafish larvae, which overexpress a stable form of β-catenin in hepatocytes, exhibited hepatocyte damage by oncogene-induced senescence and apoptosis, LPC activation, fibrosis, and differentiation of LPCs into hepatocytes, leading to the recovery of the liver. The activated LPCs persist for several days and gradually differentiate into hepatocytes. This slow progression of LPC differentiation allows the identification of small molecules that can promote the differentiation of LPCs into hepatocytes. Indeed, using this model, we discovered that treatment with EGFR inhibitors promoted LPC-driven liver regeneration, particularly the differentiation of LPCs into hepatocytes^58^. These zebrafish models will not only further help to better understand the molecular mechanisms of LPC-driven liver regeneration but also provide significant insights into promoting LPC-driven regeneration in patients with chronic liver diseases.

## Conclusions

Although LPC-driven liver regeneration occurs to restore liver parenchyma in chronic liver diseases, it does not appear to occur effectively in patients with advanced liver disease^117^. A correlation between LPC numbers and disease severity in patients with chronic liver diseases^27^ implies not only that LPCs are activated in the patients but also that the LPCs ineffectively differentiate into hepatocytes. Persistent LPCs induce inflammation and subsequent fibrosis by secreting proinflammatory cytokines^118^. Since promoting the differentiation of LPCs into hepatocytes can generate more functional hepatocytes and concomitantly reduce fibrosis, a strategy to promote the differentiation is an attractive therapeutic option for patients with advanced liver disease. To establish such a strategy, it is crucial to identify appropriate target molecules of which manipulation promotes the differentiation of LPCs into hepatocytes. Research using the animal models of chronic and severe liver injury will help to discover such molecules and eventually make the strategy feasible.
